# Requirements for implementing online information material for patients with low back pain in general practice: an interview study

**DOI:** 10.1080/02813432.2019.1569413

**Published:** 2019-01-31

**Authors:** Ditte Meulengracht Hjelmager, Line Dausel Vinther, Søren Herold Poulsen, Lone Stub Petersen, Martin Bach Jensen, Allan Riis

**Affiliations:** a Department of Development and Planning, Aalborg University, Aalborg, Denmark;; b Center for General Pracitce at Aalborg University, Aalborg, Denmark

**Keywords:** General practice, low back pain, patient education as topic, medical informatics, self-management, software design

## Abstract

**Objective:** To identify general practitioners’ (GPs) barriers and facilitators regarding the use of health information technology (HIT) in the treatment of patients with low back pain (LBP).

**Design:** A qualitative study employing a participatory design approach, with an inductive analytical thematic approach utilising semi-structured interviews. Empirical data was analysed using the qualitative data analysis software (QDAS) Nvivo.

**Setting:** General practices in Denmark.

**Subjects:** Eight interviews were conducted with an average duration of 60 min. The interviewees were GPs from different geographical settings and different organisational structures, varying in age and professional interests.

**Main outcome measures:** Barriers and facilitators for future use of the HIT application for patients with LBP.

**Results:** Through the inclusion of healthcare professionals in the design process, this study found that in order for GPs to recommend a HIT application it is essential to target the application towards their patients. Furthermore, GPs required that the HIT application should support patient self-management. Additionally, the content of the HIT application should support the initiated treatment and it should be easy for GPs to recommend the HIT application. Finally, healthcare professionals need to be involved in the design process.

**Conclusion:** When designing health IT applications for patients with LBP in general practice it is important to include both patients and GPs in the design process. GPs would be more willing to recommend a HIT application that: applies content in line with frequently used recommendations; targets patients; supports patients’ self-management; and supports the patients’ needs.

**KEY POINTS**
- Online information is currently applied in general practice to some patients with low back pain
- Online information cannot replace the GP, but can rather be a bonding tool between the patient and the GP
- It is important to address both GP and patient barriers to applying new technology and to consider the literacy level
- Participatory methods could play a central role in the future development of online information material

## Introduction

### The burden of low back pain

Low back pain (LBP) is a common condition that most people will experience at some point in their life [1]. Furthermore, LBP is the leading cause of activity loss and work absence, making it an economic burden worldwide [2]. In 2015, the Danish Health Authority estimated the annual cost of LBP to be DKK 1,820 million in Danish general practice [3]. LBP is a symptom and the specific cause of the pain is usually not identified. In a minority of cases, there are signs of specific causes such as compression of a nerve root (sciatica) or underlying serious diseases like cancers, fractures, or inflammation (red flags) [4]. However, in most cases, the cause of the pain remains unknown and it is classified as unspecific LBP [4]. LBP thus constitutes an umbrella term for a range of underlying, often unknown causes; some patients will develop persistent pain, others will be pain-free after weeks, and some will experience recurrent symptoms with periods without pain [5]. Only a minority of patients with LBP will have recovered after the first three months, with an estimated 65% of patients still experiencing pain a year after their first incident [6], thereby resulting in many general practice consultations [3]. The global burden is projected to increase; therefore, research is needed to support patients in coping with LBP in the future [7].

### Information and advice to patients with low back pain

Patient education is recommended for all patients with LBP [4,8]. According to national clinical guidelines, patient education should address education regarding health literacy, competencies, and adaptation of active behaviours [8]. Furthermore, patients should be provided with reassurance with the purpose of reducing negative beliefs and fears of illness [4,8]. In particular, patient education has the potential to have a positive effect on patients’ ability to cope with pain through, for example, elements of cognitive therapy such as focusing on positive, empathetic communication, and patients’ trust in their GP [9,10]. Delivery of patient education can, however, be time-consuming, and short consultation times underline the importance of initiatives to support the delivery of sufficient patient education in general practice [11].

### Use of the internet

During the last decade the Internet has become an effective source for education that can help engage patients in their own health [12]. Distribution of health-related information via the Internet is an inexpensive method with which to target a large group of patients [10], but the uptake of and engagement with information on the Internet greatly varies between patients [13]. In addition, GPs have expressed concerns about losing control of the treatment if they recommend HIT applications to their patients [14]. A systematic review from 2017 found it difficult to conclude what might work for whom with regard to digital self-managing interventions for LBP, and the evidence base for supporting self-management was found to be weak [15]. In one of the included studies, a high quality randomised controlled trial, tailoring and interactivity did not make a difference for patient empowerment [16]. A previous qualitative study of LBP patients’ perspectives showed the importance of including the GPs’ preferences in the design of a HIT application for the application to be effective [17]. According to this study, GPs played an essential role in the possible implementation of the HIT application as GPs are gatekeepers to secondary healthcare. The patients expressed that GPs needed to act as ‘enrollers’, applying the HIT application as a tool for dialogue and as part of their treatment plan in cooperation with patients [17].

### Aim

The aim was to identify GPs’ barriers and facilitators regarding the use of HIT in the treatment of patients with LBP.

## Material and methods

**Figure 1.**
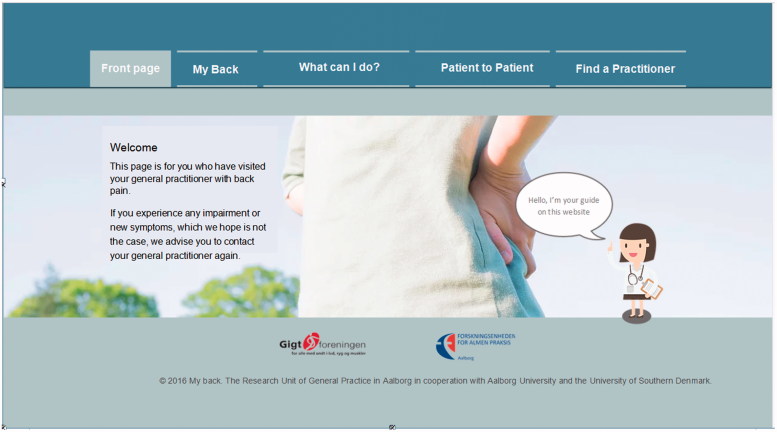
Mock-up of the online information material. *Notes:* English translation of the original mock-up with text in Danish. The mock-up was developed in cooperation with patients with low back pain.

This is a qualitative study based on participatory design (PD) methods [18]. PD is an inductive approach in which investigating, understanding, and reflecting upon the use of technologies are factors vital to inform the design process [19]. We applied semi-structured interviews with the purpose of inquiring about the GPs’ practices and their experiences of recommending HIT to their patients. In the last part of the interview respondents were presented a HIT mock-up (Figure 1) with the purpose of gaining further insights. GPs were invited to elaborate on their thoughts and ideas about a concrete source of information.

### Informants and recruitment

Based on the strong dialogue (having three interviewers from outside the field) and the narrow aim of the study, we decided on a sample size of eight GPs [20]. We included informants with maximum variation in gender, age, and type of practice. In addition, we strived to include GPs with and without special interest in LBP, with and without interests in the organisation of general practice, and with and without interests in teaching and research. Possible informants were identified by researchers and GPs at the Research Unit for General Practice in Aalborg. AR and MBJ invited them by mail or phone. Two GPs declined to participate because of high workloads. Consequently, two other GPs with characteristics similar to the declining GPs were invited; both agreed to participate.

### Data collection

The interviews took place in the GPs’ own consultation rooms to support and maintain their role as healthcare professionals in their natural work environment [21]. Two of the authors (DHM, LDV, and/or SHP) were present at each interview. The interviews were audio-recorded. In the last part of the interviews, GPs were encouraged to explore the content of different pages in the mock-up while explaining their thoughts as they did so. The GPs were asked to focus on the written content to validate the information and respond whether they felt additional information needed to be included if they were to recommend this mock-up to their patients. Suggestions regarding layout and usability were also encouraged.

**Figure 2.**
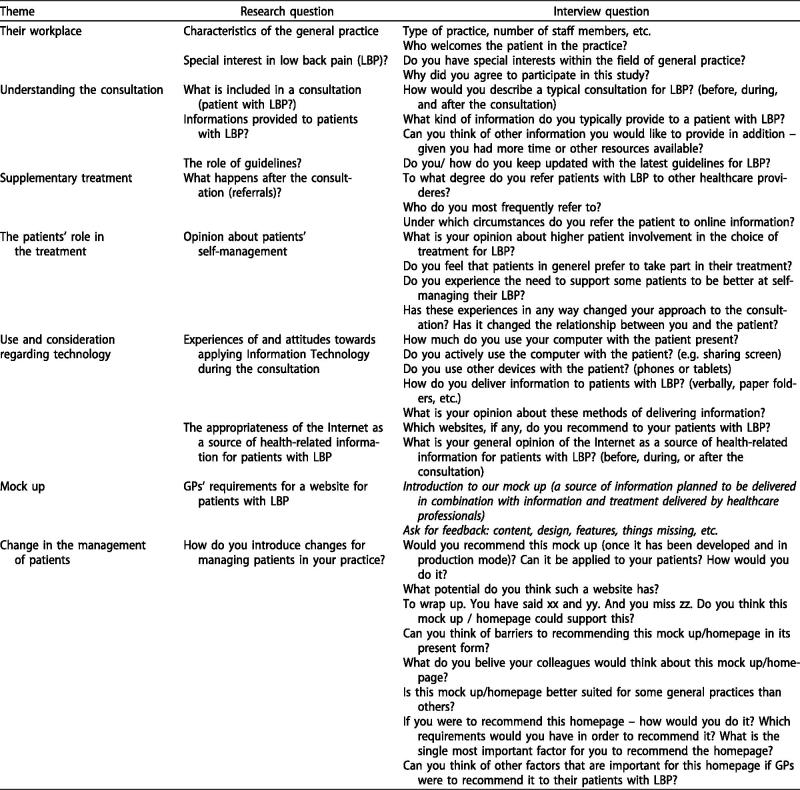
Interview guide. *Notes:* This is an English translation of the interview guide developed before the first interview. The interview guide was changed between interviews by integrating input from previous interviews.

The interview guide was developed by DMH, LDV, and SHP with inputs from all authors. The research questions in the interview guide (Figure 2) were based on the literature and experiences gathered from a previous study conducted by some of the authors of this study (DMH, LDV, MBJ, and AR) focusing on patients’ preferences for the design of online information related to LBP [17]. The interview guide was pilot tested on one GP in training. Alterations of the interview guide were performed between interviews by integrating input from one interview into the interview guide for the following interview.

GPs were all presented with the same version of the mock-up. The mock-up was developed by AR, DMH, LDV, and SHP based on input from two guideline developers (MBJ and national guideline developer (not part of the author group)), 15 patient interviews [17], and one workshop with 3 facilitators (DMH, LDV, and SHP), 1 observer (AR), and 7 participants with current or previous incidences of LBP.

### Data analysis

Audio-recordings were transcribed by DMH, LDV, and SHP and analysed using data analysis software (QDAS) Nvivo for coding and themes. We applied an inductive thematic analysis method to code units of meaning in the text and identify themes [22]. We applied value sensitive design (VSD) analysis to identify and describe values within the themes. Units of meaning in the text were identified through condensation and identification of clusters of data [23]. In adherence with the VSD analysis method, codings of the interviews were condensed into usable themes for technological design [23]. The themes were analysed to identify values that were shaped by social factors and thereby shape cultural use through interactions between technologies and users [24]. Values in this context did not refer to economic value, but rather to something a GP or a connected group found important [24].

To ensure reliability, two authors outside the field conducted the interviews (DMH, LDV, and/or SHP) and three authors (DMH, LDV, and SHP) individually coded all the interviews. Agreement was achieved by consensus in all cases. All authors agreed upon choices of themes and quotes. Quotes were chosen with the purpose to demonstrate how the findings arose from the data.

## Results

Between April and May 2017, eight one-hour interviews were conducted with GPs (Table 1). Seven GPs were recruited from the North Denmark Region, and one GP from the Central Denmark Region. In the following section themes arising from the interviews are presented.

**Table 1.** Characteristics of the GPs.

Interview #	Gender	Age	Type of practice
1	F	58	Partnership
2	F	41	Solo
3	M	47	Partnership
4	M	60	Collaborative
5	F	42	Collaborative
6	F	52	Collaborative
7	M	59	Collaborative
8	M	66	Collaborative

### Creating societal awareness of low back pain

When consulting patients with LBP, a central aspect mentioned by GPs concerns informing patients that LBP is very common, and that it in most cases LBP is not dangerous. The patients also need to be informed about which signs (red flags) to pay attention to, to support detecting potential serious illnesses as the cause of the pain. All GPs felt responsible for informing patients about the importance of their engagement in the treatment. Notably, information given to LBP patients often includes a plan to support self-management and stay active. One GP explained that she thought a HIT application could help share information concerning what LBP actually is, not only to patients but also on a societal level:

It is normal to experience back pain and it is often benign, which means that patients don’t have to restrict their activities. I sometimes wish that there was a more general understanding of back pain in society. This type of information could easily be shared through an application, I think. (GP2)

Another GP pointed to the importance of staying active despite having pain and argued that online information can be applied to support this advice:

One can argue that low back pain is part of living. It is very normal. Often I need to tell my patients that they should stay active even when having pain. In doing this, a homepage like yours, can be something I would refer to. (GP5)

GPs hence expressed that patients need to be made aware, not only that they should continue their daily activities, but how exercise therapy that strengthens the back is a central aspect of their self-management. GPs can either instruct in exercise therapy themselves or they can refer patients to physiotherapists or chiropractors. GPs argued how lack of time, resources and missing knowledge on specific exercise techniques often resulted in referrals to supplementary primary care treatment. The few GPs who actually informed patients about exercises had a special interest in musculoskeletal diseases and often used paper sheets with illustrations and instructions.

### The internet, patient skills and trustworthy health-related information

In general, GPs were positive towards the idea of using an online source of health-related information, but felt that patients needed to remain critical regarding information available online. Before referring their patients to digital information resources, GPs said that they assessed their patients’ skills and confidence in using the Internet as a source for health-related information. One described the challenges of referring to online information materials:

I think it [the Internet] is very neat, and then it is very confusing […] everyone uses it or has used it before […] it’s difficult to just send them [the patients] out on the Internet – I will not do that. I cannot vouch for what is written and I don’t know how they will interpret it. If I were to do it, I would refer to the Patient’s Handbook. I trust that. I believe there are others [professionals] who have done a proper job and I can trust the information and vouch for it. (GP4)

Potential challenges identified by the GPs were the *untamed* nature of the Internet, biased or incorrect information, losing sight of the patients and their activity online, and their inability to critically review the information they found. Since anyone can potentially contribute to information online regardless of their professional background, GPs found that patients would have difficulty in discerning accurate content from inaccurate content.

I do not recommend patients to search for health information by themselves. They just risk finding strange information provided by incompetent people. If patients directly ask me for online information, I recommend the Patient Handbook. (GP2)

One GP said that even though it is more often elderly patients he advised against using the Internet, there were also younger patients that he deemed unsuitable for applying health-related information online. He made an effort to guide his patients in their search for health-related information online:

Those who are online I try to guide. Especially the elderly who are online and get lost… You guys [the interviewers in their 20s] have this sort of a bullshit detector when you see a certain website, right? They don’t. And that’s why I want to lead them away from `Google University’ [searching via Google] and towards sites with credible sources. (GP3)

Although referring patients in general to online health-related information was common among the GPs, it was argued that only a small amount of information material relevant to LBP was available online. GPs who were not familiar with relevant online information for LBP patients expressed that it was not common to actively search for new material to present to their patients. Only if relevant material was presented to them, and preferably by a co-worker who could vouch for the material, would they consider recommending it to their patients.

If homepages with advice for back pain can be trusted. I will be happy to support their use and the education that they provided by their homepages. (GP2)

Furthermore, the integrity and reliability of a HIT application was considered important for recommending patients to access the application. Due to its open platform, YouTube was mentioned as an example of a website with low integrity. Some GPs referred to a YouTube video, giving instructions to avoid watching the following video, as it may turn out to be a randomly suggested video with no credibility. The Patient Handbook, an online resource written and maintained by Danish healthcare professionals, was regarded by some respondents as a website with high integrity and is a website that the GPs regularly refer their patients to [25]. Before vouching for a website GPs expressed that they often either read the information themselves or checked whether the source was one that they were familiar with; for instance if they knew the author. Logos and sponsors on the website should be carefully considered. GPs pointed out that they preferred HIT applications that were commercially neutral. Presenting logos on the website, of pharmaceutical companies, for instance, could for some respondents indicate that economic interests were driving the website.

### Risks related to online information

A good relationship between the patient and the GP was considered an important prerequisite for the successful delivery of information. However, the GPs expressed concern that this relationship could be impaired if digital or online information became a replacement for consulting a healthcare professional.

Empowerment does not mean that you are able to handle health issues alone by the use of the Internet. In my world, empowerment means a good relationship to your GP and the ability to discuss your problems when consulting your GP. (GP1)

The same GP acknowledged that technologies can be an important tool in healthcare but emphasised the importance of combining online information with the expertise and service of GPs. She explained:

The increase in electronic devices that replace advice and guidance from a GP can be confusing and may lead to more questions, since one unanswered question generates three new concerns. (GP1)

One concern about directing patients towards HIT applications was the risk of signalling that the GP does not have sufficient time to assist the patient, which may impair the relationship between the patient and the GP. One GP explained that he handed out information on paper because the gesture of giving his patients a physical gift works as a bonding tool in the consultation. Printing out information from a website and highlighting important information was likewise explained to be a way the GPs showed that they approved of a website:

By giving patients a piece of paper, I signal that I trust this website […] I print stuff out for my patients and mark it up. It is a great physical act that I like. It is like touching people in a way that offers some kind of bonding. Even though I would characterise my practice as reasonably electronic, it still adds something when they can take something physical away with them. (GP4)

Therefore, when designing HIT applications, it is important to consider which elements of the application can support the GP in consultation, and act as a bonding tool. During the presentation of the HIT application, GPs argued that the text was written in a condescending language, as it was too pedagogical and in some aspects too lecturing. This argument was based on the fact that GPs with their language perspective would never use that kind of phrasing. However, to what extent this critique was based on their own literacy or their knowledge of their patients is unknown, since patients had previously emphasised the need for simple and concise language [15]. This discrepancy between studies points to the need to triangulate inputs from patients and GPs, thereby enabling developers and policymakers to include both patients and healthcare professionals in the design of HIT applications.

### The added value of digital information

The GPs argued that digital information comes with both opportunities and challenges. One of the strongest arguments for using digital material was its easy accessibility. Since it would be online, GPs could refer to the HIT application verbally, or write it down for patients to access after the consultation. Assuming that they had an Internet connection, the patients could access the HIT application on whichever device they preferred, either on their PCs or tablets when they were sedentary, or on smartphones when they were on the move. Directing patients to websites instead of printing the information also presupposes that patients are able to remember the name of the website or are given a piece of paper with a link to online information.

When asked to assess the current mock-up of a HIT application, a central aspect of creating value in the consultation was whether the HIT application was able to contribute more than what a piece of paper or a paper folder offered. The paper format offers a tangible artefact with the possibility of highlighting relevant text and pictures, but is, however, static. To bring something extra to the consultation, it was argued that the HIT application should utilise the possibilities of the digital, creating a dynamic visualisation of the information. Moreover, GPs argued that animated visualisations of anatomical structures or videos explaining the functionalities of the back would serve as a helpful medium for conveying information.

Too much text alone makes reading difficult but text supported by something visual is easier to understand and remember. (GP8)

The GPs further expressed the importance of occasionally updating the HIT application with new information and features to avoid the presentation of identical material when a patient accesses the site after some time has passed – the GPs thought patients would find the application boring if the same content was presented continuously and patients would eventually dismiss the application. The need for updates was also argued as a way to sustain the integrity of the website, ensuring that the patients could access the latest information. The digital format was considered effective in this, since the provided link would always direct patients to the updated page. In contrast, regular updates of information material in paper versions were not considered feasible, since this would involve producing and handing out new material.

### Potential users of the application

While the presented HIT application was intended to target a wide variety of patients with non-specific LBP or nerve root pain, the GPs described groups of patients they would not advise to use the presented HIT application. The first group was patients with acute LBP, as the pain would likely pass in a few days and the additional information and instructions on exercises would not be relevant to them to begin with. The second group consisted of elderly patients, whom GPs deemed to have neither the technical skills nor the critical reflexivity to navigate online content successfully. Some GPs stated that this also applied to some of their younger patients.

Some people still do not own or use computers. Even down to the age of 33 years. (GP7)

One GP explained that there were patients who had neither a PC nor an Internet connection and these patients often experienced difficulties with reading and writing:

Many of my patients who are challenged in their reading skills understand a picture better than text like this. A lot of them would get lost in all that text. Instead, you could in some way visualise it, like my model [of a lumbar spine]. (GP8)

GPs expressed that patients considered suitable for the provision of online information were patients with chronic pain, patients who are already seeking information online, patients not willing or able to pay for more visits to the physiotherapist, patients willing to engage in self-management but unsure how to, and finally younger patients with uncomplicated LBP. A GP explained which patients he would recommend to use a HIT application:

Everyone who expressed that they want to perform exercises […] and then there are some who do not want to spend more money on physiotherapy. Boom! [Points at the mock-up]. Then I have some who say they want to self-manage, but are unsure what they should do. (GP3)

## Discussion

### Statement of principal findings

When designing a HIT application for patients with LBP the involvement of users is important to support the future use of the application. In this study we found that the users of the HIT applications were the GPS as well as the patients. GPs had a positive attitude towards recommending a HIT application to at least some of their patients with LBP. These patients were described as ‘people like us’ in contrast to ‘disempowered, disengaged, and disconnected’ patients. The key prerequisites for GPs to recommend an HIT application to patients were: the application needed to contain information GPs could vouch for, needed to support the initiated treatment, should be provided by a trustworthy source, and it should be easy for GPs to recommend it to patients.

### Strengths and weaknesses of the study

The carrying out of one-hour interviews in a general practice context with a sample of eight GPs with a broad variation in characteristics was a strength of this study. Furthermore, the method of using two interviewers and providing a demonstration of the mock-up further strengthened the design. The use of PD was another strength of the study. PD commits to the value of ensuring that the users of information technologies play a central part in their design [18]. Furthermore, PD is an approach in which investigating, understanding, reflection, and learning between multiple participants are factors vital to the design process [19]. GPs took on the roles of both users and designers, helping us investigate GPs’ understandings in relation to technological change. We adjusted the interview guide between interviews, but we could further strengthen the study if we had conducted the interviews in more iterations, allowing for adjustments not only to the interview guide but also to the presented mock-up between the rounds of interviews, thereby taking on a more iterative approach to integrating the GPs’ preferences into the design process.

### Findings in relation to other studies

In planning this study, we focused on acknowledging the importance of designing information content that accounted for different levels of pain and LBP symptoms. However, the division between the disempowered, disengaged, and disconnected and ‘people like us’ was not considered in the initial study design. Recent systematic reviews have also pointed to this issue when concluding that studies supporting the effect of online information have included considerably younger, higher-educated, and internet-savvy participants only [15,26].

### Meaning of the study

The consequences of not involving GPs and patients in the development of HIT applications may result in ineffective interventions which do not support the patients with the greatest need for help. Consequently, ignoring this aspect may result in eHealth systems contributing to the widening of the gap between the disempowered, disengaged, and disconnected and patients described as ‘people like us’ [27]. Providing the same content to all patients in general practice may create inequalities in its use, causing unfair or socially unjust disparities; consequently, patients should have equal access to health from their GP, no matter their personal, social, racial, or religious backgrounds [28].

### Implications for clinicians or policy makers

The provision of HIT applications can lead to higher confidence in the delivered care and in patients being more satisfied with their GP; however, to obtain maximum effect of online technologies, innovative research methods may be required [29]. Future research needs to study whether involving clinicians and patients in the development of HIT applications will be effective in supporting all patients.

## Conclusion

When developing HIT for patients with LBP in general practice it is important to include both patients and GPs in the design process. GPs would be more willing to recommend a HIT application that: applies content in line with frequently used recommendations; targets the patients; supports patients’ self-management; and supports the patients’ needs.
